# Clinical characteristics and satisfaction with the fimasartan in Korean hypertensive patients: a prospective, cross-sectional and open-label, 8-week switching study (Kanarb-hypertension epidemiology medication satisfaction study; K-HEMS study)

**DOI:** 10.1186/s40885-018-0096-2

**Published:** 2018-09-01

**Authors:** Dong Joo Oh, Su-Eun Han, Seung Hee Jeong, Myung Sook Hong, Seong Choon Choe

**Affiliations:** 1 Cardiovascular center, New Korea Hospital, Kimpo, 10086 Republic of Korea; 2 Seoul Research Institute, Boryung Pharmaceutical Co., Ltd, 136, Changgyeonggung-ro, Jongno-gu, Seoul, 03127 Republic of Korea

**Keywords:** Fimasartan, Hypertension, Medication, Satisfaction

## Abstract

**Background:**

Fimasartan (Kanarb; Boryung Pharmaceutical Co., Ltd., Seoul, Republic of Korea) is a non-protein angiotensin II receptor blocker that selectively blocks the AT1 receptor. No prior large-scale study has investigated the impact of demographics, disease, treatment, and clinical characteristics on medication satisfaction and quality of life in Korean hypertensive patients. Additionally, it is unclear whether increased medication compliance affects the achievement of hypertension treatment objectives.

**Methods:**

This was a multicenter, non-interventional, open-label and 8-week switching study. This study was divided into 2 steps. STEP I was a cross-sectional study composed entirely of hypertensive patients undergoing treatment and STEP II was a prospective observational study of hypertensive patients switching to fimasartan. A total of 12,244 and 2023 patients were analyzed in the STEP I and STEP II groups, respectively. In STEP I, we investigated demographics, clinical, disease, and treatment characteristics at the registration point and then analyzed medication satisfaction, patient compliance, and quality of life. In STEP II, the patients who switched to fimasartan were followed up for 8 weeks, and the data analyzed included changes in medication effects, satisfaction, compliance, and adverse events.

**Results:**

Some baseline characteristics, such as sex, body mass index, region of residence, educational level, and income level, affected the quality of life and medication duration in hypertensive patients. At 4 and 8 weeks, 62.5 and 69.9% of patients, respectively, reached their target blood pressure. The medication satisfaction scores were increased 4.0 ± 1.2, 5.1 ± 1.1, and 5.4 ± 1.0 at baseline, 4 weeks, and 8 weeks, respectively, and the difference was statistically significant (*p* < 0.0001). Most patients (76.4%) who changed from prior antihypertensive drug to fimasartan were not satisfied with conventional antihypertensive drugs (e.g., lack of efficacy). Among 2183 patients, 234 adverse events occurred in 151 (6.9%) and 50 adverse drug reactions occurred in 39 (1.8%).

**Conclusion:**

The demographic, clinical, disease, and treatment characteristics of hypertensive patients were investigated in this study. After switching to fimasartan, blood pressure was significantly decreased and patient satisfaction was improved. Fimasartan treatment was well tolerated and safe in hypertensive patients in Korea.

Trial registration.

**Trial registration:**

ClinicalTrials.gov Identifier: (NCT02394392).

## Background

Hypertension is a major chronic disease and a risk factor for cardiovascular diseases such as stroke, myocardial infarction, heart failure, and renal disease. The prevalence rates of hypertension in Korea were 24.6, 26.4, and 28.5% in 2007, 2009, and 2011, respectively, and continuously increasing trends have been observed in the aging population [1]. Accordingly, the number of hypertensive patients is also increasing in medical institutions [2].

Increased prevalence of hypertension has related to increased incidence of cardiovascular diseases. Approximately 35% of cerebrovascular diseases and 21% of ischemic heart diseases in Korean patients are caused by hypertension [3], and in some countries, 50% of total deaths are related to hypertension [4]. As a decrease in blood pressure in hypertensive patients can affect the incidence and death rates due to cardiovascular diseases, hypertensive patients should control their blood pressure with nonpharmacological treatments, such as lifestyle changes, combined with the use of antihypertensive drugs [5, 6].

According to, in the 2011 Korea National Health and Nutrition Examination Survey [1], the proportion of hypertensive patients over 30 years old who had controlled blood pressure (systolic blood pressure less than 140 mmHg and diastolic blood pressure less than 90 mmHg) was 36.9% for males and 49.4% for females. However, the blood pressure control rate of patients taking antihypertensive medication was 70.5% for males and 68.4% for females. This finding suggested that hypertension can be adequately managed through medication. Nonpharmacological interventions, such as improvement in lifestyle, can prevent aggravation and development in pre- or mild hypertension. If these methods are adjunctively combined with pharmacological intervention, it can reduce another cardiovascular risk. The JNC (Joint National Committee), ESH (European Society of Hypertension)/ESC (European Society of Cardiology), NICE (National Institute for Health and Care Excellence), and Korean guidelines for the management of hypertension recommended improvements in lifestyle such as exercise, smoking cessation, drinking cessation, weight loss, and diet control before or during pharmacological intervention.

However, although it is possible to control hypertension and prevent complications due to the development of many antihypertensive drugs and improvements in lifestyle, the awareness and rates of hypertension control are still low; therefore, many hypertensive patients are unaware or have a false perception that their symptoms are not related to elevated blood pressure [7].

In general, reports have shown that the health-related quality of life of hypertensive patients not diagnosed with hypertension is not different from that of normal controls. Therefore, the awareness and control rate of hypertension tend to be low, as the disease does not have a profound influence on the patient’s quality of life. However, some reports have found that many hypertensive patients do not think that their physical status is adequate [8, 9].

Due to the nature of hypertension, it’s important whether the quality of life affects the hypertension treatment outcomes. The major reason for reduced quality of life in hypertensive patients is known to be the use of antihypertensive drugs [10]. Although surveys assessing quality of life in hypertensive patients have been actively conducted in Western countries, in Korea, only one comparative study with a normal control group has been conducted [11]. In addition, although a tool for measuring quality of life was developed in Korea, large-scale studies using this tool have not been conducted.

Therefore, this nationwide, cross-sectional survey was conducted to examine blood pressure control, quality of life, and the medication satisfaction status of primarily treated hypertensive patients. In addition, the demographics, disease, treatment, and clinical characteristics affecting quality of life and blood pressure control of hypertensive patients were identified.

Moreover, although a study has been performed to assess prescription patterns associated with antihypertensive drug treatment changes in Korea, with consideration of drug ingredients and the reason for treatment discontinuation [12], no known studies have examined the reasons for prescription changes. To achieve the goal of hypertension treatment, it is necessary to maintain high drug compliance. In particular, the patient’s satisfaction with the drug can be regarded as a factor closely related to the implementation of medication guidelines and is often considered as a means of assessing treatment efficacy. No studies have investigated patient satisfaction with antihypertensive drugs. As a follow-up study of a cross-sectional survey, we investigated the reasons for prescription changes and drug satisfaction in patients who switched from antihypertensive drugs to fimasartan.

This study examined the epidemiologic data of patients with essential hypertension during treatment and investigated the treatment characteristics, such as treatment medication, medication satisfaction, and compliance, to provide basic data for adequate hypertension management. The results will help patients with hypertension to recognize the importance of blood pressure management and increase the attainment of treatment goals by increasing the interest of investigators in patient care and education.

## Methods

### Study population

Patients were considered eligible for enrollment if they met the following criteria: male or female adults aged over 20 years who signed an informed consent form and essential hypertension patients taking the same antihypertensive drug for more than 4 weeks at a screening visit in the STEP I study. Patients were excluded from STEP I if they used antihypertensive drugs for reasons other than treating essential hypertension, could not understand and complete the questionnaire, or participated in other clinical trials within 3 months.

STEP II enrolled patients from the STEP I study who had not been taking fimasartan within 3 months of the STEP I registration. Additionally, patients with drug satisfaction scores below 4 or those with scores of 5 or higher who needed to change to fimasartan treatment as determined by the investigators (i.e., lack of efficacy or compliance, occurrence of adverse events) were enrolled in STEP II.

Patients were excluded from STEP II if they had hypersensitivity to fimasartan or its ingredients, were pregnant or lactating, were receiving dialysis, had moderate to severe hepatic dysfunction, had biliary obstruction, or had a genetic disorder such as a galactose intolerance, Lapp lactose deficiency, or glucose-galactose malabsorption. As STEP I was a cross-sectional study; patients were able to stop the study at any time without stating the reason for discontinuation. Patients were withdrawn from the study if they withdrew consent to use their data, wanted to stop taking the study medication voluntarily, or wanted to stop participating in the study. Patients were also withdrawn from the study if the investigator decided that the administration of the fimasartan was not appropriate medically and ethically.

### Study design

This was a multicenter, non-interventional study. STEP I was a cross-sectional study of all hypertensive patients under antihypertensive drugs treatment. STEP II was designed as a prospective observational study of patients who switched from their antihypertensive drug to fimasartan.

Fimasartan (Kanarb; Boryung Pharmaceutical Co., Ltd., Seoul, Republic of Korea) is a non-peptide angiotensin II receptor antagonist (ARB) used for the treatment of hypertension. It is marketed in Korea and registered in 15 different countries now. This study investigated the treatment characteristics, such as the treatment drug, drug satisfaction, and compliance of essential hypertensive patients during treatment, and confirmed the basic data for appropriate management of hypertension. The purpose of the study was also to observe the change in treatment efficacy, satisfaction, and compliance when the subject was unsatisfied with the conventional drug and changed to fimasartan.

In STEP I, patients with essential hypertension who were taking an antihypertensive drug were enrolled after signing an informed consent form. At the time of enrollment, demographic information, hypertensive disease characteristics, and treatment characteristics were examined, and patient satisfaction, compliance, and quality of life were subsequently evaluated. The demographic information included sex, age, height, weight, body mass index (BMI), region of residence, educational level, and income level. The clinical characteristics included drinking, smoking, exercise, having of a home blood pressure monitor and measurement of blood pressure at home, presence of accompanying diseases (myocardial infarction, heart failure, atrial fibrillation, stroke, diabetes, and kidney disease), and family history of hypertension. If the patient’s satisfaction with antihypertensive medication was less than 4 points during STEP I or if the investigator deemed it necessary, even if the score was above 5 points, the antihypertensive drug was changed to fimasartan for STEP II. The patients were monitored up to 8 weeks at 4-week intervals, and changes in treatment effects, satisfaction, compliance, and adverse events were analyzed.

The study drug was administered according to product labeling during routine treatment. The antihypertensive drugs used to treat patients were registered in this study. All of the concomitant medications and antihypertensive drugs other than fimasartan taken by patients enrolled in STEP II during the study period were recorded on a case report form.

Fimasartan was administered to the patients enrolled in STEP II during regular treatment, and the total motivation score (forgetfulness and inattention) and total knowledge score (understanding the benefit of continuous treatment) were evaluated by the Morisky method for medication compliance evaluation on day 0 (baseline) and at 4 and 8 weeks. Total motivation scores of 0 to 1 represented low motivation and 2 to 3 represented high motivation. Total knowledge scores of 0 to 1 represented low knowledge and 2 to 3 represented high knowledge of compliance.

Fimasartan treatment information and treatment emergent adverse events after fimasartan treatment were the safety variables of this study. This study obtained relevant institutional review board approval to participate in recruitment.

### Measurements of blood pressure

Omron HEM-7220 (Omron, Tokyo, Japan) monitors were used to measure blood pressure (BP) at the study site. These were automated upper arm cuff devices based on the oscillometric method. Clinic BP was measured under standardized conditions (in the same arm by the same physician or nurse).

Target systolic BP (SBP) was < 140 mmHg, with diastolic BP (DBP) < 90 mmHg in essential hypertension [13], and target SBP was < 130 mmHg, with DBP < 80 mmHg in patients with diabetes or chronic kidney disease [14].

### Efficacy and tolerability evaluation

As this was a study of the clinical characteristics of hypertensive patients and medication satisfaction after switching to fimasartan, the evaluation parameters were as follows for STEP I and STEP II. The efficacy evaluation of STEP I included demographic characteristics (i.e., age, sex, height, weight, BMI, region of residence, educational level, and income level), clinical characteristics (i.e., drinking, smoking, exercise, blood pressure management status at home, accompanying disease and family history of hypertension), disease characteristics (i.e., blood pressure, pulse, and duration of disease), treatment characteristics (i.e., history of antihypertensive drug treatment, medication satisfaction, and medication compliance), and quality of life of hypertensive patients.

The efficacy assessments of STEP II were the quality of life of hypertensive patients, demographic/clinical/disease/treatment factors affecting quality of life, blood pressure changes after treatment with fimasartan, medication satisfaction and compliance, overall improvement, and clinical symptom evaluation by the investigators. The efficacy end points of STEP II were evaluated at 8 weeks after switching to fimasartan treatment.

The tolerability assessments of this study were the treatment information for fimasartan and the AEs after fimasartan treatment. The safety assessment was conducted in patients who had received fimasartan at least once in STEP II. The numbers and percentages of AEs, adverse drug reactions, and serious adverse events were assessed. Adverse events were coded and organized by using the Medical Dictionary for Regulatory Activities version 18.0.

### Statistical analysis

The analysis group in this study was divided into STEP I, STEP II, and safety sets. The subjects in the STEP I and STEP II analysis groups included all patients who satisfied the inclusion/exclusion criteria. The subjects in the safety set included all patients who received at least one dose of fimasartan regardless of satisfaction of the inclusion/exclusion criteria among the STEP II patients.

Descriptive statistics for the mean, standard deviation, median, minimum, and maximum values are presented for continuous variables. Frequencies and percentages are presented for categorical variables. The significance level was 5% (two-sided), and SAS software version 9.4 (SAS Institute Inc., Cary, NC, USA) was used for the analysis. Before database locking, details of the analysis were described in the statistical analysis plan.

As this was an observational study on the clinical characteristics of hypertensive patients and treatment satisfaction after changing medication to fimasartan, no hypothesis was not limited, and all variables were analyzed using descriptive statistics.

In STEP I, categorical variables such as anti-hypertensive drug and sex were summarized as frequency and percentage (%). Continuous variables such as blood pressure, age, and weight were summarized as descriptive statistics (i.e., mean, standard deviation).

In STEP II, the reasons for changing to fimasartan were summarized according to the previous antihypertensive drug type. The mean of medication satisfaction, compliance, and blood pressure were compared after changing to fimasartan. Differences after the change were analyzed with a paired t-test. Univariate logistic regression analysis was performed to determine the prognostic factors affecting quality of life/medication satisfaction and compliance in STEP I and STEP II. No imputation were used for missing data in this study.

### Sample size determination

As the purpose of this study was to investigate the clinical characteristics of essential hypertensive patients in Korea during medication treatment in STEP I and to determine the reason for a medication change and satisfaction with fimasartan in STEP II, it was necessary to determine the sample size to confirm the status. Because no previous epidemiological studies have been performed to examine the clinical characteristics of patients undergoing treatment, the sample size required for STEP II was calculated, and then the required subject numbers for STEP I were predicted. Because data on the satisfaction rate for antihypertensive drugs were lacking, the subject numbers were calculated with the assumption that prescription changes were due to dissatisfaction. Lack of efficacy, incidence of adverse events, or discomfort may lead to prescription changes, but lack of efficacy is the major reason for prescription change. According to a study on prescription changes for antihypertensive drugs, 9–14% of the patients were not responsive to the antihypertensive drug [15]. Therefore, we assumed that the median value of 12% was the drug prescription change rate and speculated that prescription changes showed a 95% confidence level and ± 1% accuracy based on the rate of 12%.

$$ \mathrm{N}=\frac{z_{a/2}^2\times p\left(1-p\right)}{d^2}=\frac{1.96^2\times 0.12\times 0.88}{0.01^2}\cong 4,056 $$

According to this formula, 4056 patients were required for the analysis in STEP II; therefore, prescription changes to fimasartan were investigated in 4500 patients with the assumption of a 10% dropout rate. Additionally, with the assumption that 1/3 of the patients who registered in STEP I may change to fimasartan, 14,000 patients were projected to enroll in STEP II.

## Results

### Patient disposition

12,691 and 2183 hypertensive patients were enrolled in STEP I and STEP II.

**Fig. 1:**
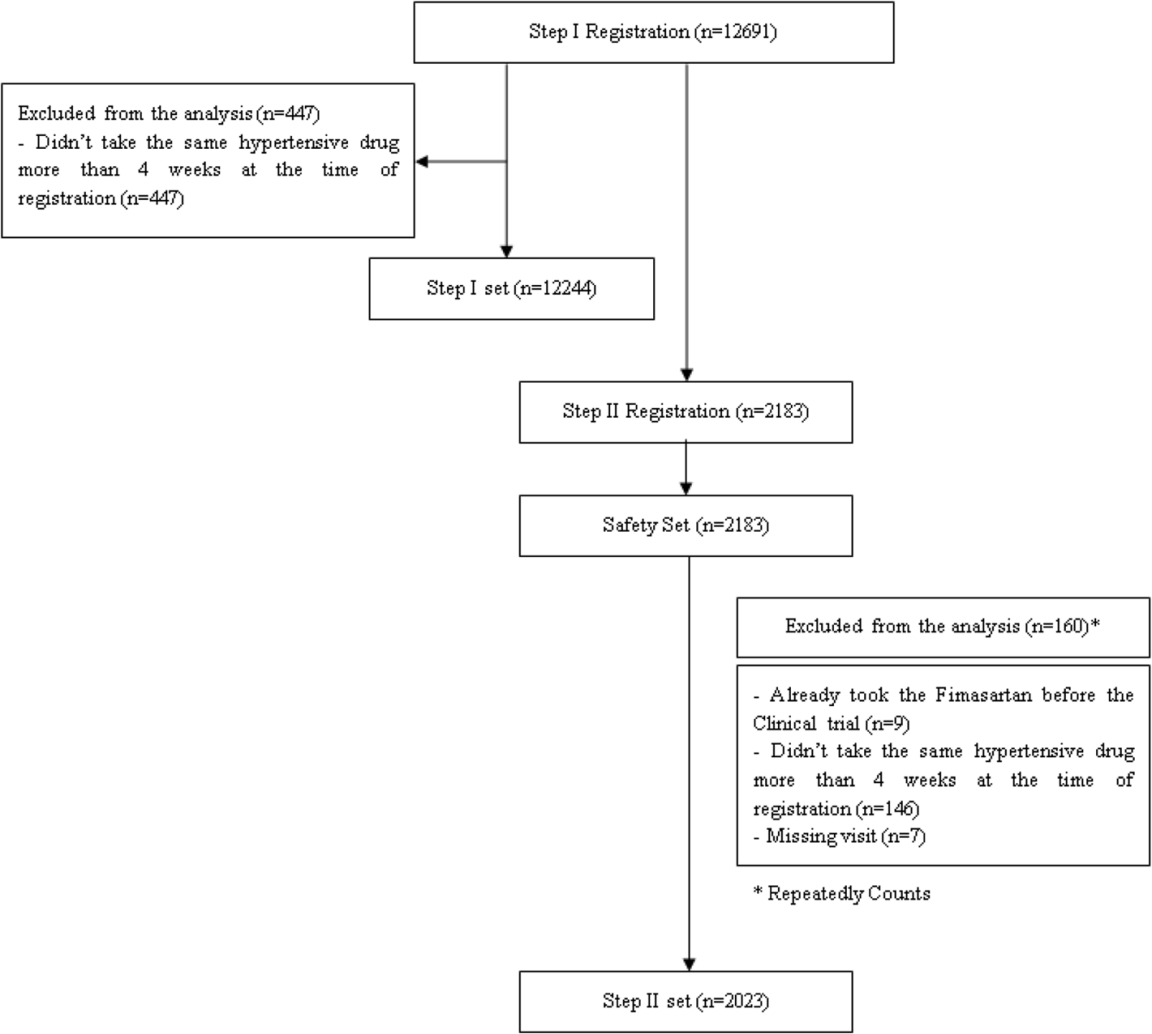
Flow chart of the study

Except for 447 who did not meet the inclusion/exclusion criteria, of the 12,691 patients enrolled in STEP I, 12,244 were included in the analysis set. Of 2183 patients registered in STEP II, excluding 160 who did not meet the inclusion/exclusion criteria or violated the protocol, 2023 were included in the analysis set. The safety set included 2183 patients among those registered in STEP II (Fig. 1, flow chart of the study).

The 447 patients who did not satisfy the inclusion criteria in STEP I were excluded because they had not received the same antihypertensive drug for more than 4 weeks at the time of enrollment. Among the 160 patients who did not meet the inclusion/exclusion criteria or violated the protocol in STEP II, 9 were excluded from the analysis due to taking fimasartan before starting the study, 146 were excluded from the analysis for not receiving the same antihypertensive drug for more than 4 weeks at the time of enrollment, and 7 were excluded from the analysis because they had missing visits.

### Baseline characteristics

The baseline demographic, clinical and disease characteristics were analyzed for 12,244 patients in STEP I. Of these, 49.9% were men and 50.1% were women. The mean age ± SD) was 61.4 ± 11.2 years. The mean height and weight were 162.4 ± 8.9 cm and 65.7 ± 11.4 kg, respectively. The mean BMI was 24.8 ± 3.2 kg/m^2^. Detailed information on region of residence, education level, and income level are described in Table 1.

**Table 1:** Patient demographics and characteristics

Characteristics	STEP I (*N* = 12,244)	STEP II (*N* = 2023)
Sex, *n*	12,244	2023
(Male, %) (Female, %)	6105, 49.86) (6139, 50.14)	(1001, 49.48) (1022, 50.52)
Age (Year)		
*n* (Mean ± SD)	12,244 (61.4 ± 11.2)	2023 (61.7 ± 11.6)
Height (cm)		
*n* (Mean ± SD)	12,163 (162.4 ± 8.9)	2013 (162.3 ± 9.0)
Weight (kg)		
*n* (Mean ± SD)	12,169 (65.7 ± 11.4)	2014 (65.6 ± 11.4)
BMI (kg/m^2^)		
*n* (Mean ± SD)	12,162 (24.8 ± 3.2)	2013 (24.8 ± 3.1)
Region of residence, *n*	12,152	1998
Metropolitan area, *n* (%)	6560 (54.0)	1139 (57.0)
Micropolitan area, *n* (%)	4466 (36.8)	724 (36.2)
Small Town, *n* (%)	1126 (9.3)	135 (6.8)
Education Level, *n*	11,919	1961
< High School graduate, *n* (%)	5304 (44.5)	882 (45.0)
≥ High School graduate, *n* (%)	6615 (55.5)	1079 (55.0)
Income level, *n*	11,953	1967
< $3500 per month, *n* (%)	7159 (59.9)	1220 (62.0)
≥ $3500 per month, *n* (%)	3520 (29.5)	502 (25.5)
Others	1274 (10.7)	245 (12.5)
Drinking, *n*	12,243	2023
Non-drinker, *n* (%)	7646 (62.5)	1298 (64.2)
Drinker, *n* (%)	4597 (37.6)	725 (35.8)
Smoking*, n*	12,243	2023
Non-smoker, *n* (%)	9273 (75.7)	1547 (76.5)
Former Smoker, *n* (%)	1195 (9.8	158 (7.8)
Smoker, *n* (%)	1775 (14.5)	318 (15.7)
Exercise, *n*	12,230	2021
Non-regularly, *n* (%)	6933 (56.7)	1245 (61.6)
Regularly, *n* (%)	5297 (43.3)	776 (38.4)
Home blood pressure monitor, *n*	12,239	2020
Without Possession, *n* (%)	8597 (70.2)	1458 (72.2)
In Possession, *n* (%)	3642 (29.8)	562 (27.8)
Blood pressure monitoring at home, *n*	3638	561
Not Measure, *n* (%)	969 (26.6)	149 (26.6)
Measure, *n* (%)	2669 (73.4)	412 (73.4)
Family history of hypertension, *n*	12,207	2010
YES, *n* (%)	5775 (47.3)	930 (46.3)
NO, *n* (%)	6432 (52.7)	1080 (53.7)
Anti-hypertensive drug prescription interval, *n*	12,150	2013
1 month, *n* (%)	6812 (56.1)	1388 (69.0)
2 months, *n* (%)	2251 (18.5)	302 (15.0)
3 months, *n* (%)	2455 (20.2)	269 (13.4)
6 months, *n* (%)	632 (5.2)	54 (2.7)
Blood pressure, *n*	11,906	2006
Systolic blood pressure (Mean ± SD, mmHg)	129.2 ± 14.2	135.7 ± 17.9
Diastolic blood pressure (Mean ± SD, mmHg)	78.6 ± 9.8	82.1 ± 11.5
Pulse rate (beats/min)		
*n* (Mean ± SD)	11,440 (73.7 ± 10.4)	1954 (73.8 ± 10.1)
Duration of hypertension (Year)		
*n* (Mean ± SD)	12,036 (7.0 ± 6.3)	1977 (7.0 ± 7.0)
Patients with Medical History, *n* (%)	3343 (27.3)	632 (31.2)
Myocardial infarction, *n* (%)	408 (3.3)	64 (3.2)
Heart Failure, *n* (%)	363 (3.0)	60 (3.0)
Atrial Fibrillation, *n* (%)	337 (2.8)	86 (4.3)
Stroke, n (%)	211 (1.7)	45 (2.2)
Diabetes Mellitus, *n* (%)	2251 (18.38)	439 (21.7)
Kidney Diseases, *n* (%)	197 (1.6)	30 (1.5)

Data represents *n* (%) as categorical variables and Mean ± SD as continuous variables

Analysis of demographic and clinical characteristics in STEP II was performed on 2023 patients who met the inclusion/exclusion criteria. The participants included 49.5% men and 50.5% women. The mean age was 61.7 ± 11.6 years. The mean height was 162.3 ± 9.0 cm, and the mean weight was 65.6 ± 11.4 kg. The mean BMI was 24.8 ± 3.1 kg/m^2^. Detailed information on region of residence, education level, and income level are described in Table 1.

Drinkers accounted for 37.6 and 35.8% in STEP I and STEP II, respectively. Non-smokers accounted for 75.7% in STEP I and 76.5% in STEP II. Detailed information on exercise, home blood pressure monitoring, family history, and prescription interval characteristics are described in Table 1.

The disease characteristics in the STEP Iwere an SBP of 129.2 ± 14.2 mmHg, a DBP of 78.6 ± 9.8 mmHg, a pulse rate of 73.7 ± 10.4 beats/min, and duration of hypertension 7.0 ± 6.3 years. In STEP II, the disease characteristics were as follows: SBP 135.7 ± 17.9 mmHg, DBP 82.1 ± 11.5 mmHg, pulse rate 73.8 ± 10.1 beats/min, and duration of hypertension 7.0 ± 7.0 years.

We also investigated the medical history of myocardial infarction, heart failure, atrial fibrillation, stroke, diabetes, and kidney disease associated with hypertension. In STEP I and STEP II, 27.3 and 31.2% had a medical history, respectively. The detailed medical history is described in Table 1.

### Efficacy

In STEP I, the total score for quality of life was 84.1 ± 18.5. The scores of each of the quality of life domains in STEP I and STEP II at baseline are described in Table 2. The total score for quality of life in STEP II at baseline was 81.3 ± 17.4. A 3-point difference was found between STEP I and STEP II at baseline. For STEP II, the total scores for quality of life at less than and more than 4 weeks were 81.1 ± 16.8 and 81.3 ± 17.4, respectively. No differences were observed between corresponding points for the total scores of quality of life in hypertensive patients.

**Table 2:** Quality of life in hypertensive patients-summary of each domain

Quality of Life assessment domains	STEP I	STEP II		
	Baseline *n* (Mean ± SD)	Baseline *n* (Mean ± SD)	4 weeks or less *n* (Mean ± SD)	Over 4 weeks *n* (Mean ± SD)
General Health Domain (GH)	12,184 (3.3 ± 1.0)	2014 (3.1 ± 1.0)	25 (3.0 ± 1.1)	1989 (3.1 ± 1.0)
Physical Domain (PD)	12,183 (16.8 ± 5.4)	2013 (16.1 ± 5.2)	25 (16.4 ± 4.9)	1988 (16.1 ± 5.2)
Mental Domain (MD)	12,183 (17.9 ± 5.4)	2014 (17.5 ± 5.1)	25 (17.6 ± 4.8)	1989 (17.5 ± 5.1)
Social Domain (SD)	12,182 (24.4 ± 4.8)	2014 (23.4 ± 4.9)	25 (22.4 ± 5.3)	1989 (23.5 ± 4.9)
Hypertension Domain (HTN)	12,181 (21.8 ± 7.0)	2013 (21.2 ± 6.4)	25 (21.7 ± 5.5)	1988 (21.2 ± 6.4)
Total Scores	12,177 (84.1 ± 18.5)	2012 (81.3 ± 17.4)	25 (81.1 ± 16.8)	1987 (81.3 ± 17.4)

In STEP II, male sex, high BMI, living in a micropolitan area instead of small town, education level greater than high school, other income categories, being a former smoker, regular exercise, possessing a home hypertension monitor, and Long-term hypertensive drug prescription interval (more than 3 months) significantly affected quality of life (Table 3). Therefore, the factors that influenced the quality of life of patients in STEP I and STEP II were comparable.

**Table 3:** Factors affecting the quality of life^a^ in hypertensive patients (STEP II)

		OR	CI	
			Lower	Upper
Sex (ref: Male)	Female	0.723	0.607	0.862
Age		1.001	0.994	1.009
BMI		1.044	1.014	1.074
Region of residence (ref: Metropolitan area)	Micropolitan area	1.095	0.908	1.320
	Small Town	0.574	0.398	0.830
Education Level (ref: < High School graduate)	≥ High School graduate	1.231	1.030	1.472
Income Level (ref: < $3500 per month)	≥ $3500 per month	1.055	0.856	1.298
	Others	1.734	1.306	2.302
Drinking (ref: Drinker)	Non-drinker	0.857	0.714	1.029
Smoking (ref: Smoker)	Non-smoker	1.196	0.938	1.523
	Former Smoker	1.613	1.097	2.373
Exercise (ref: Non-regularly)	Regularly	1.747	1.456	2.096
Home blood pressure monitor (ref: Without possession)	In Possession	1.266	1.040	1.540
Blood Pressure Monitoring at home (ref: Not Measure)	Measure	1.133	0.777	1.653
Family History of hypertension (ref: YES)	No	0.933	0.783	1.113
Anti-hypertensive drug prescription interval (ref: month)	2 months	1.178	0.917	1.514
	3 months	1.541	1.182	2.008
	6 months	4.690	2.341	9.393

^a^Satisfaction of Quality of Life (High (≥85 points) = 1, Low (< 85 Points) = 0), At this time, the high / low quality of life categorized as the top 50% / bottom 50% of the total score

*OR* odds ratio, *CI* confidence interval

Tested by logistic regression

The blood pressure changes before and after treatment with fimasartan were analyzed in STEP II. The mean SBP before and after treatment was 135.7 ± 17.9 mmHg and 128.6 ± 13.1 mmHg, respectively. The mean SBP decreased by 6.8 ± 18.4 mmHg, which was statistically significant (*p* < 0.0001). The mean DBP before and after treatment was 82.1 ± 11.5 mmHg and 79.1 ± 8.4 mmHg, respectively. The mean diastolic blood pressure decreased by 3.1 ± 11.6 mmHg, which was statistically significant (p < 0.0001) (Table 4). The target blood pressure (SBP < 140 mmHg, DBP < 90 mmHg in essential hypertension, and SBP < 130 mmHg, DBP < 80 mmHg in patient with diabetes and chronic kidney disease) was attained by 42.3% at baseline in STEP II, but the rate increased to 62.5% at 4 weeks and 69.9% at 8 weeks after treatment.

**Table 4:** Blood pressure changes after fimasartan treatment (STEP II)

Analysis Points		Systolic Blood Pressure (SBP)	Diastolic Blood Pressure (DBP)
Baseline	*n*	2006	2006
	Mean ± SD	135.7 ± 17.9	82.1 ± 11.5
End of Study	*n*	1930	1928
	Mean ± SD	128.6 ± 13.1	79.1 ± 8.4
Change (End of Study – Baseline)	*n*	1914	1912
	Mean ± SD	−6.8 ± 18.4	−3.1 ± 11.6
	*p*-value	< 0.0001	< 0.0001

Tested by paired t-test

In STEP I, the mean score of medication satisfaction for the antihypertensive drug was 5.5 ± 1.3. In STEP II, the mean score of medication satisfaction for the antihypertensive drug at baseline was 4.0 ± 1.2, but at 4 weeks after treatment, the medication satisfaction was significantly increased to 5.1 ± 1.1 (*p* < 0.0001). At 8 weeks after treatment, medication satisfaction was also significantly increased (p < 0.0001), compared to that before fimasartan treatment (Table 5).

**Table 5:** Medication satisfaction rate

Medication Satisfaction (How much did you satisfy with the hypertensive drug treatment during last 8 weeks?)	STEP I (Baseline)	STEP II (Baseline)	STEP II (4 weeks)	STEP II (8 weeks)
N	12,223	2023	1928	1827
Extremely dissatisfied, 1 point, *n* (%)	68 (0.6)	25 (1.3)	11 (0.6)	12 (0.7)
Very dissatisfied, 2 points, *n* (%)	162 (1.3)	109 (5.4)	22 (1.1)	13 (0.7)
Somewhat dissatisfied, 3 Points, *n* (%)	718 (5.9)	563 (27.8)	90 (4.7)	44 (2.4)
Neither satisfied nor dissatisfied, 4 points, *n* (%)	1881 (15.4)	855 (42.3)	392 (20.3)	244 (13.4)
Somewhat satisfied, 5 points, *n* (%)	2250 (18.4)	205 (10.1)	653 (33.9)	582 (31.9)
Very satisfied, 6 points, *n* (%)	4370 (35.8)	173 (8.6)	619 (32.1)	748 (41.0)
Extremely satisfied, 7 points, *n* (%)	2774 (22.7)	93 (4.6)	141 (7.3)	184 (10.1)
Mean ± SD	5.5 ± 1.3	4.0 ± 1.2	5.1 ± 1.1	5.4 ± 1.0
*p*-value			< 0.0001^a^	< 0.0001^b^

^a^baseline vs 4 weeks

^b^baseline vs 8 weeks

Tested by paired t-test

In STEP II, the significant factors affecting the medication satisfaction in hypertensive patients included young age, low income level, drinker, and short-term hypertensive drug prescription (Table 6).

**Table 6:** Factors affecting the medication satisfaction^a^ in hypertensive patients (STEP II)

		OR	CI	
			Lower	Upper
Sex (ref: Male)	Female	1.153	0.919	1.447
Age		0.990	0.980	1.000
BMI		0.974	0.939	1.011
Region of residence (ref: Metropolitan area)	Micropolitan area	1.142	0.892	1.463
	Small Town	1.026	0.642	1.639
Education Level (ref: < High School graduate)	≥ High School graduate	0.993	0.785	1.255
Income Level (ref: < $3500 per month)	≥ $3500 per month	0.974	0.730	1.299
	Others	0.350	0.256	0.477
Drinking (ref: Drinker)	Non-drinker	0.698	0.546	0.894
Smoking (ref: Smoker)	Non-smoker	0.916	0.664	1.262
	Former Smoker	0.924	0.560	1.523
Exercise (ref: Non-regularly)	Regularly	1.082	0.856	1.369
Home blood pressure monitor (ref: Without possession)	In Possession	0.883	0.689	1.133
Blood Pressure Monitoring at home (ref: Not Measure)	Measure	1.236	0.779	1.960
Family History of hypertension (ref: YES)	No	1.079	0.859	1.356
Anti-hypertensive drug prescription interval (ref: 1 month)	2 months	0.454	0.334	0.616
	3 months	0.303	0.225	0.409
	6 months	0.358	0.183	0.701

^a^Medication satisfaction (Above Somewhat satisfied = 1, Others = 0), Based on the medication satisfaction at 8 weeks after Baseline

*OR* odds ratio, *CI* confidence interval

Tested by logistic regression

The motivation score was 2.3 ± 1.0 at baseline. The rate of high motivation for compliance was 80.2%, and the rate of low motivation for compliance was 19.8%. At 4 weeks after switching to fimasartan, the mean motivation score for compliance was 2.6 ± 0.7. The rate of high motivation for compliance was 91.7%, and the rate of low motivation for compliance was 8.3%; these were significantly different from baseline (*p* < 0.0001). In addition, the mean motivation score for compliance after 8 weeks was 2.7 ± 0.6. The rate of high motivation for compliance was 93.5%, and the rate of low motivation for compliance was 6.6%; these were significantly different from baseline (*p* < 0.0001) (Table 7).

**Table 7:** The analysis results of the compliance rates (STEP II)

Total Scores	Baseline	4 weeks	8 weeks
	(*N* = 2021)	(*N* = 1919)	(*N* = 1818)
Motivation			
Mean ± SD	2.3 ± 1.0	2.6 ± 0.7	2.7 ± 0.6
Low (0~ 1), n (%)	401 (19.84)	160 (8.34)	119 (6.55)
High (2~ 3), n (%)	1620 (80.16)	1759 (91.66)	1699 (93.45)
*p*-value		< 0.0001	< 0.0001
Knowledge			
Mean ± SD	2.4 ± 0.7	2.5 ± 0.6	2.6 ± 0.6
Low (0~ 1), n (%)	205 (10.14)	122 (6.36)	79 (4.35)
High (2~ 3), n (%)	1817 (89.86)	1797 (93.64)	1739 (95.65)
*p*-value		< 0.0001	< 0.0001

Tested by McNemar test

The mean knowledge score was 2.4 ± 0.7 at baseline. The rate of patients with high knowledge for compliance was 89.9%, and the rate of patients with low knowledge for compliance was 10.1%. At 4 weeks after switching to fimasartan, the mean knowledge score was 2.5 ± 0.6. The rate of patients with high knowledge for compliance was 93.6%, and the rate of patients with low knowledge for compliance was 6.4%; these were significantly different from baseline (*p* < 0.0001). After 8 weeks, the mean knowledge score for compliance was 2.6 ± 0.6. The rate of patients with high knowledge for compliance was 95.7%, and the rate of patients with low knowledge for compliance was 4.4%; these were significantly different from baseline (p < 0.0001) (Table 7).

The analysis of factors affecting motivation for compliance showed that being a former smoker (OR = 3.144) and possessing a home blood pressure monitor (OR = 1.827) significantly affected motivation for compliance. The analysis of factors affecting knowledge about compliance showed that high BMI (OR = 1.140), regular exercise (OR = 1.859), and 1 month instead of 6 month of hypertensive drug prescription (OR = 0.337) significantly influenced knowledge about compliance (Table 8).

**Table 8:** Factors affecting the compliance in hypertensive patients (STEP II)

		Motivation^a^			Knowledge^b^		
		OR	CI		OR	CI	
			Lower	Upper		Lower	Upper
Sex (ref: Male)	Female	0.861	0.605	1.226	0.809	0.537	1.221
Age		1.011	0.996	1.026	0.997	0.980	1.015
BMI		1.023	0.965	1.085	1.140	1.060	1.226
Region of residence (ref: Metropolitan area)	Micropolitan area	0.727	0.497	1.065	1.304	0.791	2.149
	Small Town	0.598	0.305	1.169	0.578	0.276	1.208
Education Level (ref: < High School graduate)	≥ High School graduate	1.274	0.879	1.847	1.209	0.757	1.931
Income Level (ref: < $3500 per month)	≥ $3500 per month	1.265	0.801	1.996	1.537	0.825	2.864
	Etc.	1.234	0.675	2.258	0.961	0.479	1.928
Drinking (ref: Drinker)	Non-drinker	1.377	0.964	1.967	0.967	0.630	1.483
Smoking (ref: Smoker)	Non-smoker	1.489	0.965	2.298	0.594	0.305	1.160
	Former Smoker	3.144	1.192	8.292	0.839	0.299	2.353
Exercise (ref: Non-regularly)	Regularly	1.291	0.889	1.875	1.859	1.168	2.958
Home blood pressure monitor (ref: Without possession)	In Possession	1.827	1.161	2.875	1.628	0.976	2.715
Blood Pressure Monitoring at home (ref: Not Measure)	Measure	1.401	0.587	3.347	0.507	0.145	1.765
Family History of hypertension (ref: YES)	No	0.974	0.684	1.387	0.789	0.520	1.196
Anti-hypertensive drug prescription interval (ref: 1 month)	2 months	1.294	0.772	2.167	1.539	0.784	3.020
	3 months	5.537	2.023	15.154	1.548	0.764	3.136
	6 months	3.590	0.489	26.348	0.337	0.138	0.826

^a^Motivation: High Motivation (2~ 3) = 1, Low Motivation (0~ 1) = 0)

^b^Knowledge: High Knowledge (2~ 3) = 1, Low Knowledge (0~ 1) = 0)

OR, odds ratio; CI, confidence interval

Tested by logistic regression

In STEP II, overall improvement was assessed by the investigators. The improvement rate was 84.8% (1711/2017). Additionally, in the overall assessment of clinical symptoms, the improvement rate was 85.3% (1718/2014). (Data not shown).

The reasons for changing from a conventional antihypertensive drug to fimasartan were as follows: 76.4% of patients reported lack of efficacy, 7.9% reported adverse events, and 15.7% reported other reasons.

### Tolerability

Fimasartan was administered at least once in the 2183 patients who were included in the safety analysis. A total of 234 adverse events occurred in 151 patients (6.9%) (Table 9). The following were reported: 31 events of dizziness (1.4%, *n* = 30), 23 of headache (1.1%, *n* = 23), 17 of palpitations (0. 8%, *n* = 17), 9 of blood pressure increase (0.4%, *n* = 9), 8 of chest pain (0.4%, *n* = 8), 7 of hypotension (0.3%, *n* = 6), and 6 of cough (0.3%, n = 6). Fifty cases of adverse drug reactions occurred in 39 patients (1.8%). The most common adverse drug reactions were 10 events of headache (0.5%, *n* = 10), 9 of dizziness (0.4%, n = 9), and 7 of hypotension (0.3%, n = 6). No serious adverse events were associated with the fimasartan.

**Table 9:** Summary of the adverse events after fimasartan treatments (STEP II, Safety set)

System Organ Class Preferred Term	AEs *N* = 2183	ADRs *N* = 2183
Number of Subjects with AEs, *n* (%) [events]	151 (6.9) [234]	39 (1.8) [50]
Nervous system disorders	59 (2.7) [67]	20 (0.9) [21]
Dizziness	30 (1.4) [31]	9 (0.4) [9]
Headache	23 (1.1) [23]	10 (0.5) [10]
Syncope	7 (0.3) [7]	2 (0.1) [2]
General disorders and administration site conditions	25 (1.2) [25]	5 (0.2) [5]
Chest pain	8 (0.4) [8]	1 (0.1) [1]
Asthenia	3 (0.1) [3]	2 (0.1) [2]
Cardiac disorders	19 (0.9) [19]	2 (0.1) [2]
Palpitations	17 (0.8) [17]	2 (0.1) [2]
Respiratory, thoracic and mediastinal disorders	14 (0.6) [15]	3 (0.1) [4]
Cough	6 (0.3) [6]	2 (0.1) [2]
Investigations	11 (0.5) [11]	2 (0.1) [2]
Blood pressure increased	9 (0.4) [9]	2 (0.1) [2]
Vascular disorders	10 (0.5) [13]	7 (0.3) [9]
Hypotension	6 (0.3) [7]	6 (0.3) [7]

Adverse reactions were coded using the MedDRA version 18.0

*AE* Adverse event, *ADR* Adverse drug reaction

## Discussion

In this study, cross-sectional evaluations of the clinical characteristics of essential hypertensive patients in Korea and medication satisfaction when changing to fimasartan were investigated. In STEP I, the severity of the disease, compliance and satisfaction with medication, and quality of life during treatment were evaluated in essential hypertension patients in Korea, and demographic, clinical, disease-specific, and therapeutic factors affecting the outcome of the evaluation were identified.

In STEP II, the reasons for a prescription change were investigated in patients with essential hypertension who were not satisfied with their current antihypertensive drug and changed to fimasartan. The efficacy after treatment and changes in satisfaction and compliance at 8 weeks compared to those at baseline were analyzed.

The total score difference in the quality of life of hypertensive patients between STEP I and STEP II was approximately 3 points. In STEP II, no difference in quality of life in hypertensive patients was observed at less than 4 weeks and more than 4 weeks of the medication periods. The factors that affected quality of life were evaluated, and the trends of these factors in STEP I and STEP II were comparable.

In STEP II, the changes in blood pressure after treatment with fimasartan were investigated. The SBP and DBP were significantly decreased after fimasartan treatment. In addition, the percentages of patients reaching target blood pressure at 4 weeks and 8 weeks were 62.5 and 69.9%, respectively. The percentage of patients reaching target blood pressure increased as treatment progressed.

The medication satisfaction scores in STEP II were 4.0 ± 1.2 before fimasartan treatment (baseline), 5.1 ± 1.1 at 4 weeks, and 5.4 ± 1.0 at 8 weeks, and the satisfaction levels significantly increased as treatment continued. The factors that significantly influenced medication satisfaction in STEP I included young age, living in a micropolitan area, high income level, being a smoker, non-regular exercise, lack of a home blood pressure monitor, and short-term hypertensive drug prescription. In STEP II, the factors that significantly influenced medication satisfaction were young age, low income level, drinking, and short-term hypertensive drug prescription.

In the compliance analysis (Morisky method), the motivation and knowledge scores at 4 and 8 weeks were significantly higher than at baseline. The factors that significantly influenced the motivation for compliance included being a former smoker and having a home blood pressure monitor. For the knowledge of compliance, high BMI, regular exercise, and 1 month of hypertensive drug prescription were significant factors.

In the final analysis of STEP II, the overall improvement rate was 84.8%, and the overall rate of improvement of clinical symptoms was 85.3%. Most of the symptoms improved. The reason for switching from an antihypertensive drug to fimasartan in STEP II was reported as a lack of efficacy by 76.4% of patients. Switching antihypertensive drugs to fimasartan did reduce the risks of blood pressure-related cardiovascular disease in hypertensive patients.

All of the adverse events were reported on the drug labels.

## Conclusion

The results of this study demonstrated that patient satisfaction with medication improved when they switched from a conventional antihypertensive drug to fimasartan. Patients with a short-term prescription showed greater satisfaction. Additionally, blood pressure was significantly decreased after switching to fimasartan, and fimasartan administration was well tolerated and safe in hypertensive patients in Korea.
